# Genomic Insights into Disease Resistance in Sunflower (*Helianthus annuus*): Identifying Key Regions and Candidate Genes for *Verticillium dahliae* Resistance

**DOI:** 10.3390/plants13182582

**Published:** 2024-09-14

**Authors:** Yue Yu, Jianfeng Yang, Jian Zhang, Loren H. Rieseberg, Jun Zhao

**Affiliations:** 1 Department of Botany and Biodiversity Research Centre, University of British Columbia, Vancouver, BC V6T 1Z4, Canada; yue.yu@ubc.ca; 2 College of Horticulture and Plant Protection, Inner Mongolia Agricultural University, Hohhot 010010, China; jianfengyang89@126.com (J.Y.); zhj19890128@imau.edu.cn (J.Z.)

**Keywords:** sunflower, *Verticillium dahliae*, disease resistance, GWAS, Verticillium Wilt, crop biotic resistance, resistant cultivars, membrane vesicles

## Abstract

Sunflower (*Helianthus annuus*) is a globally significant field crop, and disease resistance is crucial for ensuring yield stability and crop quality. *Verticillium dahliae* is a notorious soilborne pathogen that causes Verticillium Wilt (VW) and threatens sunflower production worldwide. In this study, we conducted a comprehensive assessment of sunflower resistance to *V. dahliae* across 231 sunflower cultivar lines, from the Sunflower Association Mapping (SAM) population. We employed EMMAX and ridge regression best linear unbiased prediction (rrBLUP) and identified 148 quantitative trait loci (QTLs) and 23 putative genes associated with *V. dahliae* resistance, including receptor like kinases, cell wall modification, transcriptional regulation, plant stress signalling and defense regulation genes. Our enrichment and quantitative real-time PCR validation results highlight the importance of membrane vesicle trafficking in the sunflower immune system for efficient signaling and defense upon activation by *V. dahliae*. This study also reveals the polygenic architecture of *V. dahliae* resistance in sunflowers and provides insights for breeding sunflower cultivars resistant to VW. This research contributes to ongoing efforts to enhance crop resilience and reduce yield losses due to VW, ultimately benefiting sunflower growers and the agricultural sector.

## 1. Introduction

Sunflower (*Helianthus annuus*) is one of the most significant oilseed crops globally, contributing to approximately 20 million tons of oil and over 22 million tons of confectionery consumption annually [1]. *Verticillium dahliae* is reported to infect over 200 plant species, including major crops like sunflower, tomato (*Solanum lycopersicum*), potato (*Solanum tuberosum*), and others [2,3,4,5,6,7,8].

Sunflowers infected by *V. dahliae* are reported in major production regions worldwide, including Ukraine, Russia, the European Union, Argentina, China, and the United States [3,9,10]. The resting structures of *V. dahliae*, microsclerotia, can persist in soil for years and rapidly germinate to infect host sunflowers through their roots. Vascular transport enables the pathogen to colonize inside the plant, leading to leaf chlorosis, necrosis, wilting, or death at the reproductive stage of plants [11,12,13]. The symptoms of Verticillium Wilt (VW) can cause yield losses exceeding 30% in susceptible cultivars, resulting in substantial economic impacts on agricultural production. Therefore, researching resistance strategies and breeding resistant sunflower varieties against *V. dahliae* is critical for maintaining sunflower production [14,15,16].

To date, the use of fungicides on plants infected with *V. dahliae* has been largely ineffective at stemming VW [17,18]. However, there is considerable evidence of genetic resistance against *V. dahliae* in sunflower [19] and other plant species, including cotton [20] (*Gossypium arboretum*), tomato [21], potato [22], and many more [23,24]. The resistance mechanisms that have been identified to date are similar to those known to be deployed against other fungal pathogens [24]. These include extracellular enzymes like chitinase, which can digest *V. dahliae* cell walls [25], and defense-related proteins such as polygalacturonase inhibitors [26] that protect plant cell walls by impeding pectin digestion. In addition, pathogen-associated molecules such as chitin and *V. dahliae* effector proteins are recognized by various receptor-like proteins [27,28], triggering immune responses. These include the activation of key signal transduction pathways, such as the jasmonic [29] and salicylic acid [30] pathways, as well as the reprogramming of genome-wide transcription [24]. In sunflower, Guo et al. [19] compared the transcriptomes of two cultivar lines (resistant and susceptible) using RNA-Seq and revealed some differentially expressed genes in plant–pathogen interaction pathways.

In the present study, we phenotyped the cultivated Sunflower Association Mapping (SAM) population for resistance to *V. dahliae* and carried out a comprehensive genome-wide association study (GWAS) to investigate the genetic architecture of resistance in sunflowers. The SAM population comprises 288 cultivated lines that encompass over 90% of the genetic diversity in cultivated sunflower [31,32]. It has been widely used in previous GWAS studies on abiotic and biotic stress resistance, including resistance to drought, flooding, salt and nutrient stress, and to downy mildew and stem canker [33,34,35,36,37,38]. Studies of downy mildew and stem canker successfully revealed complex polygenic genetic architectures and identified the quantitative trait loci (QTL) underlying resistance to these diseases [37,38], suggesting that the SAM population offers a promising means of investigating sunflower biotic stress resistance. Here, we conducted genome-wide association (GWA) analyses with both single-nucleotide polymorphisms (SNPs) and presence–absence variants (PAVs) derived from whole-genome sequencing of the SAM population [37] to achieve the following specific objectives: (1) to evaluate *V. dahliae* resistance in the SAM population; (2) to investigate the genetic architecture of *V. dahliae* resistance in cultivated sunflowers with both marker types; (3) to identify the quantitative trait loci (QTL) and candidate genes underlying sunflower resistance to *V. dahliae* and highlight their likely functions; and (4) to uncover the possible defense mechanisms in resistant sunflower accessions. Our ultimate goal is to use this information to guide the breeding of resistant sunflower cultivars. 

## 2. Materials and Methods

### 2.1. Materials

#### 2.1.1. Plant Materials

We obtained 287 SAM population lines from the National Plant Germplasm System (https://www.ars-grin.gov/Collections#plant-germplasm, accessed on 1 March 2020), which is operated by the USDA-ARS. Of these, only 231 lines successfully germinated and produced sufficient replication for downstream analyses. Additionally, two sunflower varieties, JK601 (resistant) and LD5009 (susceptible), from China, were included as checks. The SAM accessions have been categorized based on their agronomic usage and breeding history into the following groups: HA nonoil, HA oil, RHA nonoil, RHA oil, non-oil-introgressed, landrace, and open-pollinated varieties (OPVs). HA (maintainer lines) typically refers to unbranched plants, while RHA (restorer lines) refers to branched plants. The introgressed category includes oil and nonoil types with a history of wild *H. annuus* introgression. The landrace category comprises Native American landraces, and the OPV category represents a stage of pre-hybridization in sunflower breeding [32].

#### 2.1.2. Fungal Materials

The *V. dahliae* strain V-89 was isolated from a single diseased plant, collected from the main sunflower growing region in China (Wuyuan County, Inner Mongolia). This strain was initially purified and cultured on PDA (Potato Dextrose Agar) medium, and then extensively propagated on wheat bran medium for 20 days. Lastly, the conidia were washed with sterile water and adjusted to a final concentration of 1 × 10^7^ conidia per mL.

#### 2.1.3. Genomic Data

Whole-genome sequencing raw data for the SAM population were generated by Hübner et al. [37], with an average coverage of 5–25× per line. Raw reads were aligned to the latest sunflower reference assembly HA412v2 reference genome (https://sunflowergenome.org/, accessed on 17 October 2022), from which the following two sets of markers were generated.

Single-Nucleotide Polymorphism (SNP) variants were called with methods described by Todesco et al. [39], following these steps. Raw reads with low-quality sequences and adapters were trimmed with Trimmomatic v0.36. Cleaned reads were then aligned to the reference genome using NextGenMap v0.5.3. Variant calling was performed with the Genome Analysis Tool Kit after excluding transposable elements. To eliminate low-quality variants, the variant quality score recalibration used 20 samples with the highest coverage as a goldset, then hard filters were applied to retain only bi-allelic SNPs with 90% tranche, a minor allele frequency greater than 0.03 and a genotyping rate above 50%. Finally, SNPs were phased using a linkage map with Beagle 5.0 with default settings [40]. The final set of filtered SNPs comprised 3,699,248 biallelic variants.

Presence–absence variants (PAVs) were generated as described by Lee et al. [41]. Briefly, the raw sequencing data described above were aligned to the HA412v2 genome using BWA mem (0.7.12). Reads with a mapping quality less than 10 were removed using Samtools (1.9). For each 100 bp window, the sequencing depth was measured at every position using Samtools with a custom perl script. Window depths equal to or above zero were translated into a genotype file of 0’s and 1’s, respectively. After filtering to retain PAVs with a minor allele frequency >5%, 7,541,946 PAVs were used for downstream analyses.

### 2.2. Methods

#### 2.2.1. Pathogen Inoculation

The conidial root inoculation method was used for the evaluation of sunflower resistance to *V. dahliae* [42]. Six or seven seeds of each line were sown in small pots with a diameter of 13 cm containing sterilized soil and covered with sand. After seed germination, five seedlings were kept in each pot, and five pots were prepared for each line. The pots were placed randomly in a greenhouse with the temperature controlled between 25 °C to 28 °C and a relative humidity of 50% to 70%. After four true leaves had developed (V4 stage), which was reached approximately 21 days post sowing, the seedlings were inoculated with a conidial suspension of *V. dahliae* (1 × 10^7^ conidia/mL) using the root inoculation as described in Peng et al. [42]. The experiment was set up with 3 replicates. After the susceptibility check (LD5009) showed typical VW symptoms of leaf necrosis, the disease scales (0~4) were scored for each plant based on the following criteria [43]: 0 = no symptoms, 1 = wilting and stunting < 25%, 2 = wilting and stunting ≥ 25%, 3 = wilting and stunting ≥ 50%, 4 = wilting and stunting ≥ 75%. The wilting and stunting percentage represents the number of necrotic leaves relative to all leaves.

For each accession, three replicates with a total of 25 plants were scored. The disease index (DI) was then calculated for each accession as follows:

*Disease index* = [((0 × *N*_0_) + (1 × *N*_1_) + (2 × *N*_2_) + (3 × *N*_3_) + (4 × *N*_4_))/(*N*_1_ + *N*_2_ + *N*_3_ + *N*_4_)] × 100

Nn = number of plants in each scale.

In each trial, the susceptible (LD5009) and resistant (JK601) cultivars were included as checks. Therefore, to reduce the error among different trials, a correction disease index (DIcorr) was employed:

*DIcorr* = *DI* − (*DIn*, *controls* − *DI total*, *controls*)

DIn, control = mean of the susceptible check in each trial; DI total, control = mean of the susceptible checks across all trials.

#### 2.2.2. GWA Mapping Using EMMAX

The efficient mixed-model association eXpedited (EMMAX) model [44] was used for the GWA of both SNPs and PAVs. The Shapiro–Wilk test and Box–Cox transformation were applied to the corrected disease index (DIcorr) to improve normality to meet the EMMAX model assumptions for GWA. EMMAX (emmax-intel64-20120205) is a mixed model that can reduce false positive results by incorporating two covariates, kinship (K-matrix) and population structure (P-matrix). Both were calculated based on SNP data for the 231 accessions used in our analyses and then added to the final GWA model for the SNP and PAV datasets, respectively. The K-matrix was calculated using emmax-kin and the P-matrix was calculated based on the first three principal components (PCs) estimated from a PLINK pruned SNP set with the following parameters: 50 kbp sliding window, 50 bp steps and r^2^ threshold of 0.2 [45]. The log-transformed *p*-value (−log_10_P) indicates marker significance. The Bonferroni significance threshold was initially implemented for multiple testing correction to identify significant associations. The Bonferroni-corrected 5% significance threshold (−log_10_P) for 3,699,248 SNPs was 7.9 and for 7,541,946 PAVs was 8.1.

#### 2.2.3. Identification of Candidate Markers and QTLs

**Figure 1:**
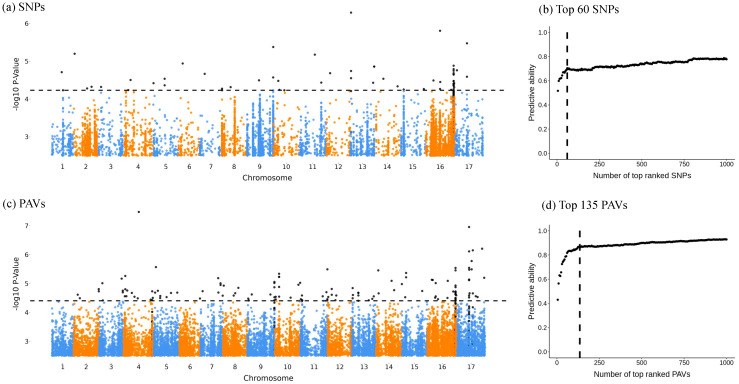
Genome-wide association mapping results for 3,699,248 single-nucleotide polymorphisms (SNPs) and for 7,541,946 presence–absence variants (PAVs). Manhattan plot of SNPs (**a**) and PAVs (**c**) with −log_10_P > 2.5; each point represents a single marker. Candidate marker threshold in black dashed horizontal lines, and candidate markers within QTLs highlighted as black points; non-candidate markers are in orange and blue. Changes in predictive ability for top markers identified with EMMAX (high −log_10_P) for SNPs (**b**) and PAVs (**d**). From top five to top 1000 markers (increments of five) using rrBLUP. Candidate marker threshold in black dashed vertical lines.

The Bonferroni significance threshold can be overly conservative when many tests are conducted [46], such as in a GWA analysis with millions of SNPs or PAVs. For a polygenic trait, this may mean that few or no QTLs exceed the significance threshold. A complementary approach is to implement ridge-regression best linear unbiased prediction (rrBLUP) for top-ranking markers based on GWA *p*-values [34]. This approach examines aggregated marker effects on trait predictive ability [47]. Here, we tested the aggregate effects of the 5–1000 top markers on the predictability of each trait using rrBLUP (v4.6.3), with a step of five markers in each test. Candidate markers were subsequently selected based on when the improvement in the predictive ability started to significantly decrease (Figure 1b,d). The kin.blup() function in the rrBLUP package [47,48] was employed to solve the BLUP model using an additive relationship matrix calculated from testing markers with the A.mat() function. To estimate the predictive ability, the 231 samples were randomly divided into a testing set of 162 (70%) samples and a validation set of 69 (30%) samples. The predictive ability was calculated as the correlation between the actual and predicted trait values in the validation set. This process was repeated 500 times for a final average predictive ability. The candidate markers selected by rrBLUP were then extended into high LD haploblocks using the PLINK (v1.9) clump command with --clump-kb, 250; --clump-r^2^, 0.4; --clump-range-border 5 [45]. All the above analyses were run independently on both the SNP and PAV datasets. The clumped candidate regions for both marker types were independent and were then merged into a single list of QTLs. Genes within 5 kbp of the identified QTL areas were our final “candidate genes”.

#### 2.2.4. From Candidate Regions to Genes and Functions

To understand how the identified candidate genes might contribute to plant–pathogen interactions and the plant defense mechanism, we used the functional annotations of 46,223 genes predicted from the latest sunflower reference genome HA412v2 [49]. The predicted genes were annotated with InterProScan version 5.0 [50] from the following three databases: protein family database (Pfam [51]), InterPro and gene ontology (GO).

#### 2.2.5. GO and KEGG Enrichment Analyses

For GO enrichment, we clustered annotations at three levels of biological organization: biological pathways (BP), molecular functions (MF), and cellular components (CC). This was accomplished using the topGO package in R version 4.2.0 [52,53]. The statistical tests employed a classic algorithm, Fisher’s exact test, and an FDR correction with a significance threshold of 0.1 for multiple testing. KEGG enrichment was performed using the clusterProfiler package in *R* version 4.4.0 [54] on all candidate genes, with the parameters set to a pvalueCutoff of 0.05, a qvalueCutoff of 0.2, and pAdjustMethod “BH”. All genes were annotated to KEGG pathways through the KEGG Automatic Annotation Server (KAAS; https://www.genome.jp/kegg/kaas/, accessed on 5 April 2024 [55]).

#### 2.2.6. Experimental Validation through Quantitative Real-Time PCR

Five lines from each of the three resistance levels (immune, resistant and susceptible) and a set of promising candidate genes were selected for experimental validation. The selected immune lines (DIcorr = 0) were SAM 136, SAM 158, SAM 163, SAM 177, and SAM 201. The resistant lines (0 < DIcorr < 25), selected from both high and medium treatment resistance levels, included SAM 001, SAM 010, SAM 014, SAM 078, and SAM 100. The susceptible lines (DIcorr > 25) were SAM 088, SAM 132, SAM 175, SAM 184, and SAM 086. Three replicates from each line were germinated. Three days after root development, the treatment group was inoculated with a conidial suspension of *V. dahliae* (1 × 10^7^ conidia/mL) using the root inoculation method [42], while the control group was inoculated with water. Roots were collected at five different time points (0, 1, 3, 5, 7 dpi) for RNA extraction. Total RNA was extracted using TRIzol reagent (Invitrogen, Waltham, MA, USA) and purified with a PureLink RNA Mini Kit (Ambion, Austin, TX, USA) following the manufacturer’s instructions. RNA was reverse transcribed using SuperScript III reverse transcriptase (Invitrogen, Carlsbad, CA, USA) and oligo(dT) primers to obtain cDNA. Quantitative real-time PCR (qRT-PCR) was performed using SYBR^®^ Premix Ex Taq™ II (Tli RNaseH Plus, Kusatsu, Japan) according to the manufacturer’s protocol (Takara: DRR420A). The qRT-PCR parameters included an initial denaturation step at 95 °C for 10 min, followed by 40 cycles of 95 °C for 15 s and 60 °C for 1 min. The sunflower Actin gene (*HaActin*) served as an endogenous control to normalize the expression levels of the candidate genes. The relative transcript levels were calculated using the 2^−∆∆CT^ method for three independent biological replicates [56].

## 3. Results

### 3.1. Evaluation of Disease Resistance of Sunflower Cultivar Lines

The corrected disease index (DIcorr) categorized cultivar lines into five resistance levels, ranging from immunity to high susceptibility (classification summarized in Table 1; detailed information in Table S1). The check lines JK601 and LD5009 exhibited DIcorr values of 9.24 (highly resistant, HR) and 42.91 (highly susceptible, HS), respectively. Among the SAM accessions, the majority showed high to medium resistance to *V. dahliae*, with 102 and 103 accessions falling into these two categories. Conversely, only eighteen lines displayed full immunity, while eight accessions were classified as having medium susceptibility.

### 3.2. GWA Mapping and Identification of Top Candidate Markers and QTLs

The raw corrected disease index (DIcorr) was not normally distributed when tested by a Shapiro–Wilk normality test (*p* value = 6.622 × 10^−7^, Figure S1). The Box–Cox transformation of DIcorr using the EnvStats package [53,57] brought the Shapiro–Wilk *p*-value to 0.0034 (Figure S1). Although still deviating from normality, the transformed DIcorr dataset better fit the normal distribution compared to the raw DIcorr; thus, the transformed DIcorr was used for GWA mapping.

No markers passed the strict Bonferroni-corrected 5% significance threshold (−log_10_P) for either SNPs (Figure 1a) or PAVs (Figure 1c). Thus, we performed rrBLUP analyses to select candidate markers [34]. The predictive ability started to decrease when the −log_10_P from EMMAX output approached 4.232, resulting in 60 SNPs selected as candidate markers, with a DIcorr predictive ability of circa 0.7 (Figure 1b; Table S2). Similarly, the turning point of −log_10_P is at 4.404 for PAV data resulting in a candidate set of 135 PAVs, which explain over 85% of DIcorr variation (Figure 1d; Table S2). We identified 107 candidate regions for PAVs and 41 regions for SNPs. These were subsequently collapsed into 148 QTLs in total, which were distributed across all chromosomes (Figure 1; Table S3).

### 3.3. From Candidate Regions to Genes and Functions

We extracted 51 unique candidate genes from 34 QTLs (Table S4), as not all 148 QTLs captured genes within a 5 kbp window. Among our candidate genes, 23 putative genes were associated with plant disease resistance, classified into five functional categories: receptor-like kinases, cell wall modification enzymes, transcription regulators, plant stress signaling and defense regulation genes (Table 2). These putative genes were distributed across 16 unique QTLs, with notable accumulation on chromosomes 4, 5, 9 and 11. 

Putative genes (Table 2) included three receptor-like genes: two Leucine-Rich Repeat kinases (LRR) and one gene in the THH1/TOM1/TOM3_domain. The enzyme category included four genes, three of which were annotated to pectin metabolism, ubiquitin-conjugating enzyme and GDP-fucose protein O-fucosyl transferase on chromosome 9. Another gene on chromosome 4 was identified as a plant glutathione S-transferase (GST). We identified two Pentatricopeptide Repeat (PPR) genes, which can serve as transcription regulators that mediate gene expression in plastids or mitochondria to support plant resistance [58,59]. The signaling category included eight genes, comprising two protein kinase domain genes and one Serine/threonine-protein kinase (STK) gene within 5 kbp of QTL: chr11:195944400..195944400. Additionally, there was one gene each for the following functions: the WD40 repeat domain, Ankyrin repeat domain, IQ Calmodulin-binding motif (IQM), Trichome Birefringence-Like (TBL) proteins, and host signal transduction and stress response domain. Lastly, we found six candidate genes related to defense regulation. Three genes were annotated to the Zinc finger domain, being involved in plant–pathogen interactions [60]. Two genes, Arf/Sar-related proteins in the small GTPase superfamily, are important regulators for generating and trafficking proteins and antimicrobials through vesicles, essential for intracellular signaling and regulation [61,62,63]. The last gene in this category is annotated to the CASP C-terminal domain, the C-terminal region of Golgi membrane proteins, which also plays a crucial role in vesicle transportation and membrane trafficking [63,64].

### 3.4. Enrichment Analysis

GO and KEGG enrichment analyses were conducted with 51 candidate genes. GO enrichment included genes that were annotated with at least one GO term relative to the global HA412v2 database of annotated GO terms. Due to the limited number of candidate genes, no Molecular Function (MF) or Biological Process (BP) categories showed any significant enrichment (Table S5). However, the Golgi membrane (GO: 0000139) in the Cellular Component (CC) was marginally enriched with an adjusted *p*-value of 0.06 and the ko00514 pathway for O-glycan biosynthesis, which is an important modulator for the plant defense process [65], was significantly enriched, with an adjusted *p*-value of 0.03. 

### 3.5. Experimental Validation

**Figure 2:**
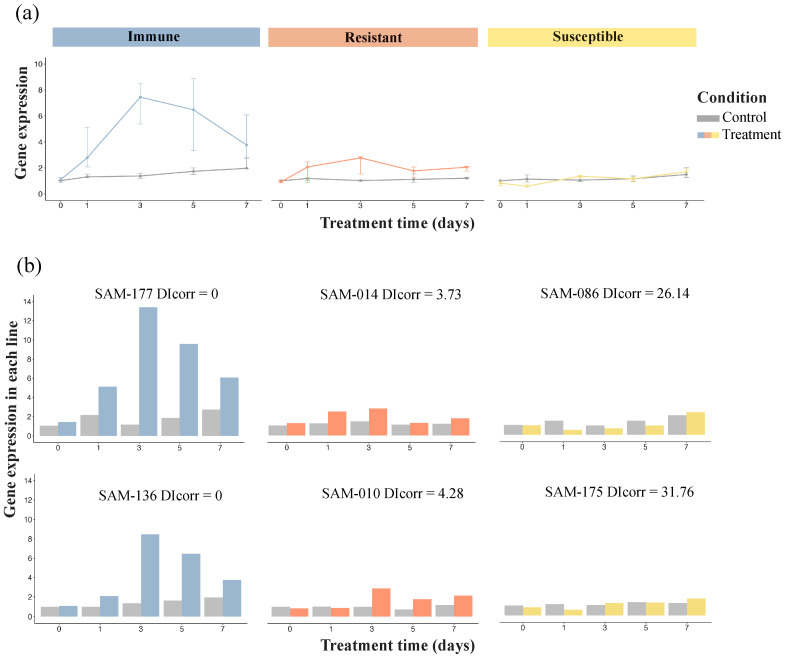
Gene expression of Ha412HOChr08g0327051 before and after *Verticillium dahliae* inoculation over time using quantitative real-time PCR in three resistance categories: immune, resistant and susceptible. Results for all sunflower lines in each category (**a**) and for two selected sunflower lines in each category (**b**). *X*-axis represents the treatment time: 0, 1, 3, 5 and 7 dpi. *Y*-axis represents the gene expression levels. Different colors represent different group conditions: grey for control group, and blue, orange and light yellow for treatment in immune, resistant and susceptible lines, respectively. (**a**) Median gene expression data for five tested lines in each resistance category for the control and treatment group. Points represent the median gene expression levels at each time point, and the error bars extending from each point indicate the interquartile range, with the lower bound representing the 25th percentile and the upper bound representing the 75th percentile of the gene expression levels. (**b**) Paired gene expression in selected line over time. DIcorr stands for corrected disease index.

We selected 11 genes out of 51 candidate genes for validation experiments using q-PCR (Table S6). Our rationale for drawing from the larger candidate gene pool is that genes without annotation or with unclear and indirect annotation might also reveal signals of *Verticillium dahliae* resistance through validation experiments. After artificial inoculation, candidate gene Ha412HOChr08g0327051 showed a consistently low expression in all control groups, regardless of the resistance category. A significant variation in expression was observed among treatment groups: immune lines had the highest expression, followed by resistant lines, while susceptible lines showed little difference between the treatment and control groups (Figure 2a). In immune lines SAM 177 and SAM 136, gene expression increased dramatically after initial treatment, peaking at 13.42 and 8.48 at 3 dpi, respectively, then decreased gradually with the prolongation of the inoculation time (Figure 2b). Resistant lines SAM 014 and SAM 010 presented a similar pattern, with a much lower peak expression at 3 dpi. Susceptible lines SAM 086 and SAM 175 also showed lower expression levels compared to those of the controls at 1 dpi. These results suggest that gene Ha412HOChr08g0327051 is involved in *V. dahliae* resistance and highlights that the first three days post-inoculation are likely to be critical for the activation of resistance genes in sunflowers. The remaining tested 10 candidate genes showed inconsistent expression patterns between the control and treatment groups at different inoculation timepoints (Table S6), indicating that these candidates likely do not contribute to the sunflower resistance against VW. 

## 4. Discussion

*Verticillium* wilt causes substantial damage to sunflower production worldwide, as management practices such as fungicide sprays or crop rotations struggle to control the disease [17,18,66]. Therefore, we approached this problem from a different direction by investigating the genetic basis of resistance to *V. dahliae*. We first phenotyped 231 genetically diverse sunflower cultivar lines following the inoculation of the *V. dahliae* pathogen and calculated a disease index. The evaluation results showed that 18 accessions were fully immune to *V. dahliae* (DIcorr = 0, Table S1), or about 7% of all examined SAM accessions; however, when highly resistant accessions (0 < DIcorr ≤ 10) were included, this percentage increased to over 50%. We also compared our results to previous studies of resistance to biotic stress in the SAM population. Among the 18 fully immune lines, we found that four accessions were also highly resistant to stem canker [38] (Table S1) and 10 accessions were immune to downy mildew [37] (Table S1). Three accessions, SAM 054 (PI-655014), SAM 067 (PI-561918) and SAM 177 (PI-5999782), have common immunity for all three types of disease. This summarized information aids sunflower breeding programs in selecting lines with overall high resistance towards multiple major common pathogens in the field.

Genome-wide association analyses of 231 SAM accessions revealed 135 candidate PAVs, 60 candidate SNPs, 148 QTLs, and 51 candidate genes. These signals were found across almost all chromosomes (Figure 1, Table 2), suggesting the polygenic architecture of sunflower *V. dahliae* resistance. This finding is not surprising as many QTLs were also found for powdery mildew and stem canker resistance [37,38], and disease resistance clusters have been found on all sunflower chromosomes [67]. In our study, the contribution of many loci with small effects may explain the lack of signals above the strict Bonferroni threshold, but these loci are captured by rrBLUP due to its ability to quantify cumulative marker effects [48].

Among the 148 QTLs, 16 unique QTLs harbored 23 putative *V. dahliae*-resistant genes. The GWA signals for these primary QTLs were captured by either SNP or PAV markers (Figure 1). Notably, three QTLs on chromosome 9 and 11 harbored over one third (9 out of 23) of putative genes (Table 2). Two QTLs located on both ends of chromosome 11 (Ha412HOChr11:12496400..12496400 and Ha412HOChr11:195944400..195944400) captured five putative genes, including LRR, Zinc finger domain, protein kinase domain and STK. A similar pattern was observed in a 84.5 kbp candidate QTL on chromosome 9 (Ha412HOChr9:199587300..199671800), which captured a total of four genes related to LRR and enzymes responsible for pectin metabolism, protein degradation and glycan biosynthesis with a significant KEGG enriched signal (Table S5).

The identified 23 putative *V. dahliae*-resistance genes fall into the five main categories of mechanism involved in plant resistance to *V. dahlia*, as reviewed by Song et al. [24]: receptor-like kinases, cell wall modification enzymes, transcription regulation, plant stress signaling and defense regulation. Receptor-like kinases such as LRR proteins are important cell-surface receptors for pathogen recognition, as well as for the activation of downstream plant defenses against fungal pathogen invasions [28,68]. The enzymes involved in pectin metabolism, protein degradation and glycan biosynthesis play key roles in plant defense via cell wall modification [26,69]. Additionally, transcriptional regulator PPR mediates gene expression in plant organelles, further enhancing plant defense systems [58,59]. Plant stress signaling functions include serine/threonine-protein kinases (STKs), which contribute to resistance via both pathogen perception and through the transmission of external pathogen signals via phosphorylation [70,71]. The WD40 repeat domain can convert extracellular signals into intracellular chemical defense responses [72]. Although the salicylic acid or jasmonic acid-mediated signal transduction found by Gao et al. [73] was not directly captured in our candidate genes, we found other signaling functions such as IQ Calmodulin-binding motif (IQM), which is suggested to positively regulate jasmonic acid biosynthesis and plant defense [74] (Table 2). Our identified candidate genes can also aid the future construction of a gene co-expression network in sunflower biotic resistance processes [75].

Lastly, we found that membrane vesicle trafficking is likely an important defense mechanism in sunflowers. Two putative genes at the beginning of chromosome 8 and at the end of chromosome 16 (Ha412HOChr08g0327051 and Ha412HOChr16g0793171) were annotated to the ARF/SAR-related protein in the Small GTPase superfamily. One of these (Ha412HOChr08g0327051) was validated in our q-PCR experiment, with strong evidence of expression responses to *V. dahliae* in tested immune lines. The predicted protein for this tested gene encodes an important regulator for generating and trafficking vesicles, essential for the efficient and timely transportation of proteins and antimicrobials for intracellular and extracellular use, thus aiding in signaling and defense actions in the plant immune system in order to fight pathogen infections [61,62,63]. It should also be highlighted that the defense function of small GTPase can be interfered with by effectors secreted by resistant pathogens to enhance self-replication and in turn increase virulence [76,77]. Vesicle trafficking processes can involve several endomembrane organelles, such as the endoplasmic reticulum (ER), Golgi apparatus and others [78]. Our GO enrichment result is also in line with the role played by the vesicle trafficking process in resistance, highlighting that the Golgi membrane (GO:0000139) as an enriched cellular component in *V. dahliae* resistance (Table S5). 

## 5. Conclusions

In conclusion, our study elucidates the genetic basis of *V. dahliae* resistance in sunflowers using a genome-wide association study, offering valuable insights for breeding disease-resistant cultivars. By evaluating *V. dahliae* resistance in 231 sunflower association mapping accessions, we identified 18 lines with full immunity to *V. dahliae* and three lines showing shared immunity to VW, stem canker, and downy mildew. Genome-wide association analyses using EMMAX and rrBLUP with both SNPs and PAVs confirmed the polygenic architecture of *V. dahliae* resistance in cultivated sunflowers. We also identified 148 QTLs and 23 putative *V. dahlia*-resistant genes within these QTL regions, classified into five functional categories: receptor-like kinases (LRR family and others), cell wall modification enzymes (including pectinesterase), transcription regulation (PPR), plant signaling (STK, IQM and others) and defense regulation (mainly small GTPase). Validation experiments and GO enrichment analyses further imply that membrane vesicle trafficking plays an important role in sunflower resistance, although other mechanisms likely contribute as well. This research advances our understanding of the plant–pathogen interaction between sunflower and *V. dahliae* and provides valuable information for breeding resistant sunflower cultivars.

## Figures and Tables

**Table 1:** Number of sunflower association mapping (SAM) accessions classified into evaluation level based on corrected disease index (DIcorr).

Resistance Level	Corrected Disease Index (DIcorr)	Number of SAM Accessions
Immunity (I)	DI = 0	18
High Resistance (HR)	0 < DI ≤ 10	102
Medium Resistance (MR)	10 < DI ≤ 25	103
Medium Susceptibility (MS)	25 < DI ≤ 40	8
High Susceptibility (HS)	DI > 40	0

**Table 2:** Twenty-three candidate plant disease resistance genes were classified into five functional categories: receptor-like kinases, enzymes, transcription regulation, signalling and defense regulation. The gene function in each resistance category, annotated gene name, position and each gene’s related quantitative trait loci (QTL) are presented in order; for more functional descriptions, see Table S4.

Resistance Classification	Resistance Gene Function	Annotated Ha412 Gene				Quantitative Trait Loci (QTLs)	
		Name	Chromosome	Start	End	Range	Length (kbp)
Receptor-like kinases	Leucine-rich repeat (LRR) receptor-like protein kinases domain	Ha412HOChr09g0430181	9	199,673,064	199,676,740	Chr9:199587300..199671800	84.501
		Ha412HOChr11g0485191	11	12,489,907	12,491,965	Chr11:12496400..12496400	0.001
	THH1/TOM1/TOM3_domain	Ha412HOChr05g0201031	5	2,135,315	2,140,825	Chr05:2142873..2142873	0.001
Enzymes	Plant glutathione S-transferases (GSTs)	Ha412HOChr04g0161611	4	54,865,558	54,867,010	Chr04:54869161..54869161	0.001
	Pectinesterase	Ha412HOChr09g0430121	9	199,621,706	199,624,213	Chr9:199587300..199671800	84.501
	Ubiquitin conjugating enzyme E2	Ha412HOChr09g0430131	9	199,654,317	199,657,697	Chr9:199587300..199671800	84.501
	GDP-fucose protein O-fucosyl transferase	Ha412HOChr09g0430141	9	199,661,153	199,666,513	Chr9:199587300..199671800	84.501
Transcription regulator	Pentatricopeptide Repeat (PPR)	Ha412HOChr05g0201051	5	2,144,526	2,145,764	Chr05:2142873..2142873	0.001
		Ha412HOChr13g0624831	13	159,187,929	159,189,605	Chr13:159192700..159192700	0.001
Signalling	WD40 repeat domain	Ha412HOChr04g0149911	4	12,506,690	12,515,912	Chr4:12515100..12515100	0.001
	Ankyrin repeat domain	Ha412HOChr04g0193401	4	209,950,163	209,950,678	Chr4:209869900..209957500	87.601
	Signal response regulator receiver domain	Ha412HOChr05g0212311	5	47,237,986	47,241,942	Chr5:47234800..47234800	0.001
	IQ Calmodulin binding motif (IQM)	Ha412HOChr07g0294431	7	34,499,627	34,502,094	Chr07:34502762..34502762	0.001
	Trichome Birefringence-Like (TBL) proteins	Ha412HOChr08g0326731	8	385,886	387,955	Chr08:391677..391677	0.001
	Protein kinase domain	Ha412HOChr11g0531821	11	195,939,617	195,939,979	Chr11:195944400..195944400	0.001
		Ha412HOChr11g0531831	11	195,940,158	195,946,483	Chr11:195944400..195944400	0.001
	Serine/threonine-protein kinase (STK)	Ha412HOChr11g0531841	11	195,949,268	195,951,584	Chr11:195944400..195944400	0.001
Defense regulation	Small GTPase superfamily ARF/SAR related protein	Ha412HOChr08g0327051	8	812,081	815,534	Chr08:815128..815128	0.001
		Ha412HOChr16g0793171	16	190,225,952	190,245,150	Chr16:190241501..190241501	0.001
	CASP C-terminal domain	Ha412HOChr17g0837651	17	106,817,961	106,823,156	Chr17:106827000..106827100	0.101
	Zinc finger domain	Ha412HOChr01g0016781	1	73,395,376	73,396,787	Chr01:73392344..73392344	0.001
		Ha412HOChr11g0485201	11	12,494,087	12,496,285	Chr11:12496400..12496400	0.001
		Ha412HOChr16g0802371	16	212,155,400	212,161,057	Chr16:212158239..212187958	29.720

## Data Availability

All code associated with this project is available at: https://github.com/yueyu27/Verticillium-dahliae-resistance-in-cultivated-sunflowers, accessed on 3 August 2024.

## Supplementary Materials

The following supporting information can be downloaded at: https://www.mdpi.com/article/10.3390/plants13182582/s1, Figure S1: Frequency plot and QQ plot testing for data normality for raw corrected disease index (DIcorr) and boxcox transformation of DIcorr. Table S1: DIcorr recorded for 231 sunflower accessions in this study, classified into four resistance levels. Including metadata of accession name, line name, class, package number, sunflower association mapping (SAM) population. Overlapping immune lines for verticillium wilt, stem canker, and downy mildew are identified. Table S2: Predictive ability change along the top ranked single nucleotide polymorphisms (SNPs) and presence–absence variants (PAVs). Table S3: Information on candidate marker and QTLs identified with both SNPs and PAVs: including candidate marker position, QTL positions and lengths, genes within 5 kbp window of QTL areas. Table S4: Annotated Pfam, Interpro, GO terms, and gene positions for 51 candidate genes. Resistance classification, gene function, function description, related QTLs and references for 23 putative genes for *V. dahliae* resistance. Table S5: GO and KEGG enrichment results. Table S6: Information and results on validation experiments using quantitative real-time PCR (q-PCR) in 11 tested candidate genes. Including tested gene names, q-PCR reaction system, condition, designed primers and gene expression results.
