# Supplementary material for: Genomic Insights into Disease Resistance in Sunflower (Helianthus annuus): Identifying Key Regions and Candidate Genes for Verticillium dahliae Resistance
Source: Plants (Basel). 2024 Sep 14;13(18):2582. doi: 10.3390/plants13182582 (PMC11434647; doi:10.3390/plants13182582)
Supplement: Supplementary file 1 [file plants-13-02582-s001.zip › SI_FigureS1_2024Sep13.pdf]

Supporting Information (SI)

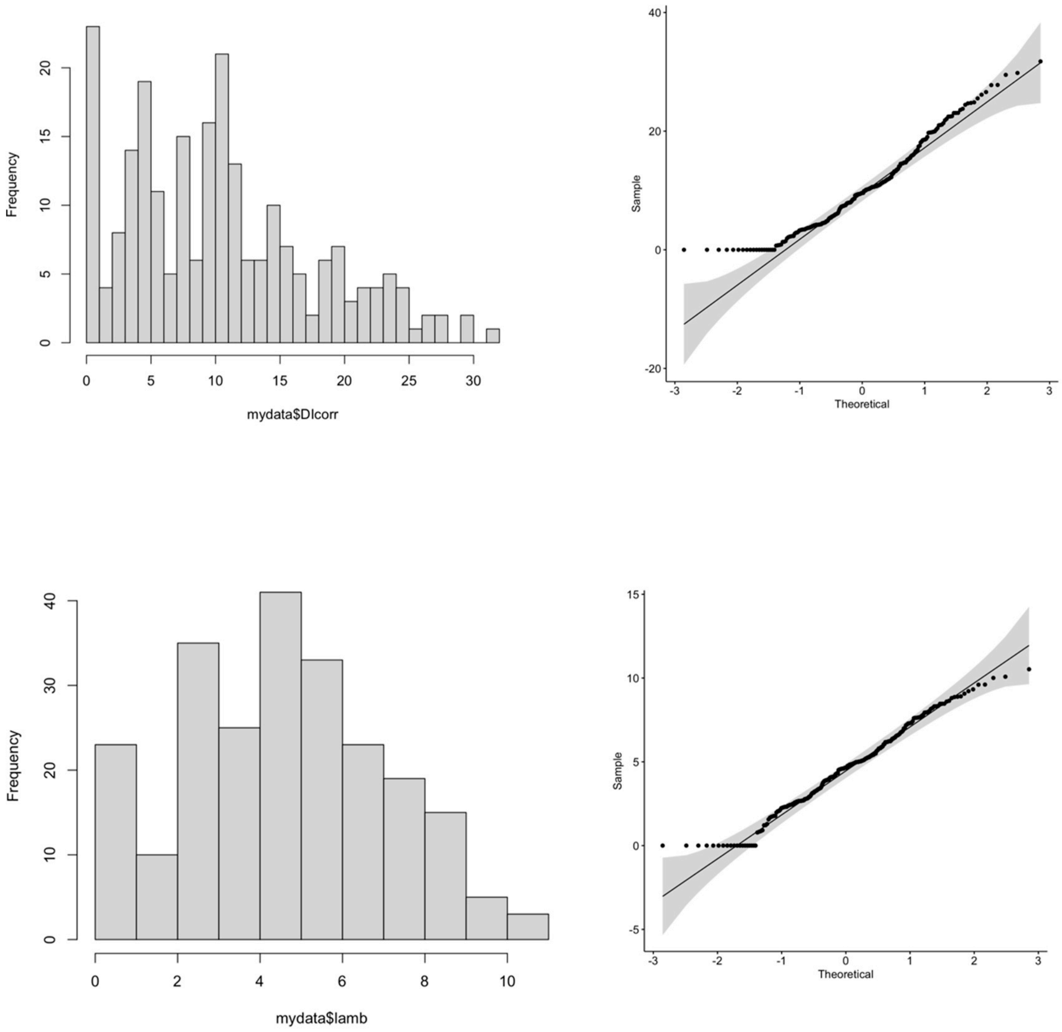

Figure S1. Frequency plot and QQ plot testing for data normality for raw DIcorr (top two) and boxcox transformation of DIcorr (bottom two).
